# Lamotrigine ethanol monosolvate

**DOI:** 10.1107/S2056989018005819

**Published:** 2018-04-19

**Authors:** Charlie L. Hall, Jason Potticary, Hazel A. Sparkes, Natalie E. Pridmore, Simon R. Hall

**Affiliations:** a School of Chemistry, University of Bristol, Cantock’s Close, Bristol, England BS8 1TS, England

**Keywords:** crystal structure, lamotrigine, ethano­late

## Abstract

Lamotrigine is an active pharmaceutical ingredient used as a treatment for epilepsy and psychiatric disorders. Single crystals of an ethano­late solvate, C_9_H_7_Cl_2_N_5_·C_2_H_5_OH, were produced by slow evaporation of a saturated solution from anhydrous ethanol. Within the crystal structure, the lamotrigine mol­ecules form dimers through N—H⋯N hydrogen bonds involving the amine N atoms in the *ortho* position of the triazine group. These dimers are linked into a tape motif through hydrogen bonds involving the amine N atoms in the *para* position. The ethanol and lamotrigine are present in a 1:1 ratio in the lattice with the ethyl group of the ethanol mol­ecule exhibiting disorder with an occupancy ratio of 0.516 (14):0.484 (14).

## Abstract 2

The main motif within the structure is a lamotrigine dimer stabilized by two ethanol mol­ecules. Here the lamotrigine dimer forms using amines in the *ortho* position of the triazine group.

## Chemical context   

Anti­convulsants are a group of drugs used principally in the treatment of epilepsy, which have also been shown to aid in the treatment of psychiatric conditions such as bipolar disorder. Although the drugs are effective when inside the body, many suffer from having low solubility and bioavailability. Prime examples of such drugs are carbamazepine (Uzunović *et al.*, 2010 ▸), phenytoin (Widanapathirana *et al.*, 2015 ▸) and lamotrigine (Vai­thia­nathan *et al.*, 2015 ▸), which are all categorised as BCS (biopharmaceutical classification system) class II (low solubility, high permeability).

In an attempt to increase the solubility of BCS class II drugs, extensive studies have been undertaken to produce crystal structures including the active pharmaceutical ingredients (APIs) with lower crystal lattice energies. In the case of lamotrigine, Cheney *et al.* (2010 ▸) investigated the solubility of 10 novel forms, including salts, co-crystals and solvates, showing the possibility of creating many stable lamotrigine compounds. The structures of lamotrigine co-crystals and solvates are stabilized due to the large number of hydrogen bonds that can form with the 1,2,4-triazine-3,5-di­amine group.
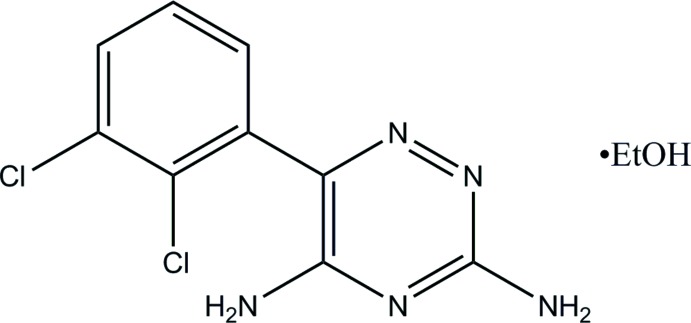


In this work, the structure for the ethano­late (I), previously only obtained as a powder pattern (Garti *et al.*, 2008 ▸), is defined. This new structure determination affords a deeper insight into the different hydrogen-bonding networks that can form in the lamotrigine crystal.

## Structural commentary   

**Figure 1:**
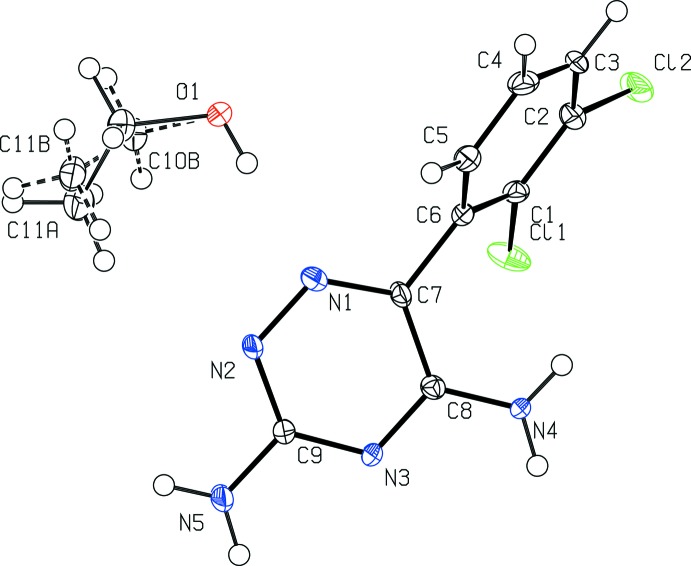
A displacement ellipsoid plot of (I), showing the atom-labelling scheme. Displacement ellipsoids are drawn at the 50% probability level.

**Figure 2:**
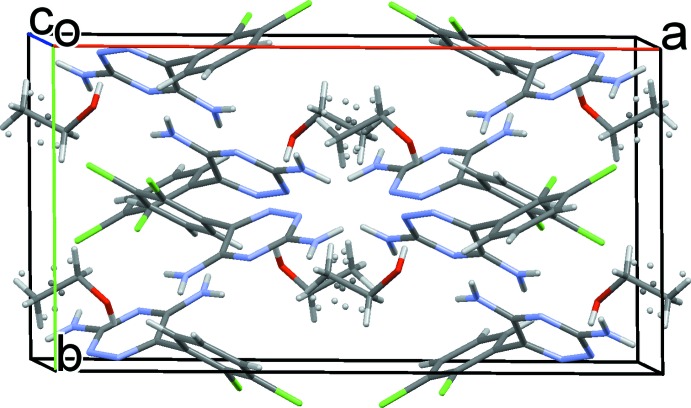
The crystal packing of (I), viewed along the *c* axis.

A displacement ellipsoid plot for lamotrigine ethano­late is shown in Fig. 1 ▸. The central dihedral, C1—C6—C7—C8, sits at an angle of 63.5 (9)°, the flexibility of which allows for the inclusion of solvent mol­ecules to form hydrogen-bonding networks. Central dihedral angles for lamotrigine solvates are included in Table 1 ▸. Fig. 2 ▸ shows the unit cell for (I), which consists of eight lamotrigine mol­ecules and eight ethanol mol­ecules. The main motif within the structure is a lamotrigine dimer stabilized by two ethanol mol­ecules. Here the lamotrigine dimer forms using the amine N atoms in the *ortho* position of the triazine group.

## Supra­molecular features   

**Figure 3:**
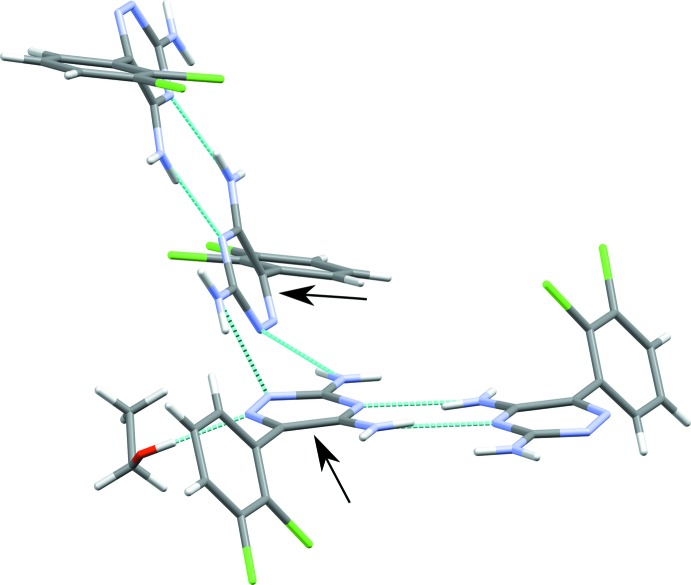
The bonding motif of adjacent lamotrigine dimers. The angle between the dimers was calculated using the planes of the indicated triazine rings.

In the crystal, adjacent in-plane lamotrigine dimers are linked *via* hydrogen bonding of the amines in the *para* position of the triazine group (Table 2 ▸). Each dimer sits at an angle of 67.2 (5)° to the next closest dimer, measured with respect to the in-plane triazine rings, highlighted in Fig. 3 ▸.

## Database survey   

**Figure 4:**
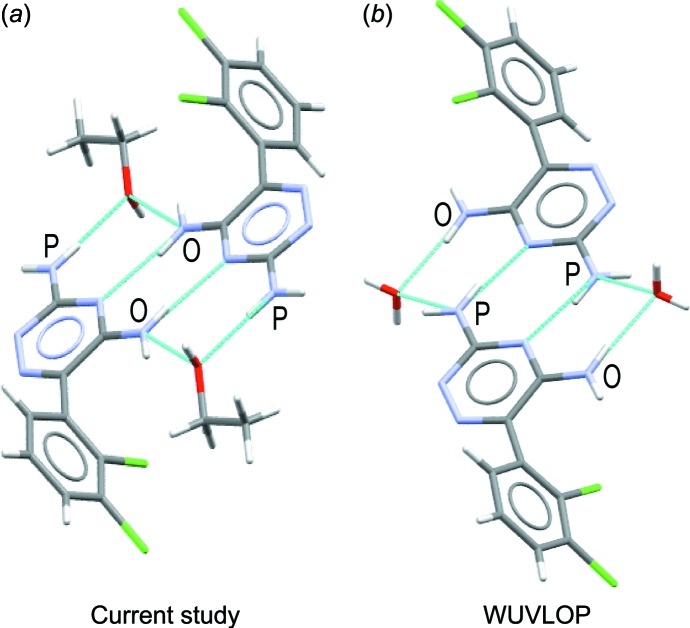
(*a*) The dimerization motif in (I), held together with the amines in the *ortho* position of the triazine group. The amine in the *ortho* and *para* positions are labelled with O and P, respectively. (*b*) The dimerization motif in the ethano­late hydrate structure, held together with the amines in the *para* position of the triazine group.

A database survey of the Cambridge Structural Database (CSD, version 5.38, last update May 2017; Groom *et al.*, 2016 ▸) showed a list of 35 existing co-crystal/solvate structures for lamotrigine, including 6 structures incorporating alcohols, but no ethanol solvate. The most similar structure compositionally to (I) is the ethanol solvate monohydrate (Cheney *et al.*, 2010 ▸); however, the arrangement contrasts quite dramatically, with the dimer formation of the lamotrigine mol­ecules using the amine N atoms in the *para* position, shown in Fig. 4 ▸. This change in dimerization motif leads to a reduction in density of the lamotrigine ethano­late over the lamotrigine ethanol monohydrate by 5%.

Analysis of the previously published lamotrigine alcohol solvates shows a trend between the alcohol chain length and whether the lamotrigine dimers form on the *ortho* or *para* group of the triazine. The two densest structures are the methanol disolvate (Hanna *et al.*, 2009 ▸) and the ethanol solvate monohydrate, where lamotrigine dimers are connected *via* the amines in the *para* position of the triazine. Conversely, the methanol monosolvate (Janes *et al.*, 1989 ▸), iso­propanol solvate (Qian *et al.*, 2009 ▸) and title compound form dimers from the amine on the *ortho* positions. The least dense structure is the butan-1-ol solvate monohydrate (Sridhar & Ravikumar, 2011 ▸), which has similar arrangement to the dense structures, with the dimers held apart by the large butanol solvent mol­ecules. The densities of the lamotrigine structures are highlighted in Table 1 ▸.

## Synthesis and crystallization   

Lamotrigine (>98%, Acros Organics) was saturated in a solution of pure anhydrous ethanol (>99.5%, Sigma Aldrich) over several weeks. Crystals of lamotrigine ethano­late were produced *via* slow evaporation of 1 ml of the solution over 72 h.

## Refinement   

Crystal data, data collection and structure refinement details are summarized in Table 3 ▸. All of the hydrogen atoms were located geometrically (aromatic C—H = 0.95 Å, methyl C—H = 0.98 Å, ethyl C—H = 0.99 Å, O—H = 0.84 Å N—H= 0.88 Å) and refined using a riding model [aromatic, ethyl and amine *U*_iso_(H) = 1.2 times parent atom *U*_eq_, methyl and alcohol *U*_iso_(H) = 1.5 times parent atom *U*_eq_]. The ethanol solvent in the lattice is disordered over two positions; the occupancies of the two positions were refined with the sum set to equal 1, refining to give relative occupancies of 52:48. Restraints (SIMU 0.01 0.02) were applied to maintain sensible thermal displacement parameters for the carbon atoms.

## Supplementary Material

Crystal structure: contains datablock(s) I. DOI: 10.1107/S2056989018005819/fy2126sup1.cif

Structure factors: contains datablock(s) I. DOI: 10.1107/S2056989018005819/fy2126Isup2.hkl

Supporting information file. DOI: 10.1107/S2056989018005819/fy2126Isup3.cml

CCDC reference: 1826282

Additional supporting information:  crystallographic information; 3D view; checkCIF report

## Figures and Tables

**Table 1:** Chosen parameters for the comparison of lamotrigine alcohol solvates

Structure	Central dihedral angle (°)	Dimerization motif	Density (g cm^−1^)
Methanol disolvate	63.7 (2)	*para*	1.50
Ethanol monohydrate	67.6 (0)	*para*	1.49
Methanol monosolvate	80.1 (5)	*ortho*	1.45
Ethanol solvate (I)	63.5 (9)	*ortho*	1.42
2-Propanol solvate	69.6 (8)	*ortho*	1.36
Butan-1-ol solvate monohydrate	71.2 (1)	*para*	1.34

**Table 2:** Hydrogen-bond geometry (Å, °)

*D*—H⋯*A*	*D*—H	H⋯*A*	*D*⋯*A*	*D*—H⋯*A*
O1—H1*A*⋯N1	0.84	2.01	2.848 (7)	179
N4—H4*A*⋯N3^i^	0.88	2.10	2.972 (7)	172
N4—H4*B*⋯O1^ii^	0.88	2.14	2.841 (7)	137
N5—H5*A*⋯O1^iii^	0.88	2.16	3.014 (7)	163
N5—H5*B*⋯N2^iv^	0.88	2.14	2.987 (8)	161

Symmetry codes: (i) 
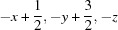
; (ii) 
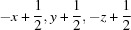
; (iii) 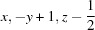; (iv) 
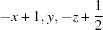
.

**Table 3:** Experimental details

Crystal data	
Chemical formula	C_9_H_7_Cl_2_N_5_·C_2_H_6_O
*M*_r_	302.16
Crystal system, space group	Monoclinic, *C*2/*c*
Temperature (K)	100
*a*, *b*, *c* (Å)	21.2458 (15), 10.2320 (8), 14.8428 (11)
β (°)	118.808 (4)
*V* (Å^3^)	2827.3 (4)
*Z*	8
Radiation type	Mo *K*α
μ (mm^−1^)	0.46
Crystal size (mm)	0.39 × 0.25 × 0.13

Data collection	
Diffractometer	Bruker APEXII CCD
Absorption correction	Multi-scan (*SADABS*; Bruker, 2015 ▸)
*T*_min_, *T*_max_	0.602, 0.745
No. of measured, independent and observed [*I* > 2σ(*I*)] reflections	21376, 2925, 2634
*R*_int_	0.053
(sin θ/λ)_max_ (Å^−1^)	0.629

Refinement	
*R*[*F* ^2^ > 2σ(*F* ^2^)], *wR*(*F* ^2^), *S*	0.098, 0.234, 1.41
No. of reflections	2925
No. of parameters	193
No. of restraints	48
H-atom treatment	H-atom parameters constrained
Δρ_max_, Δρ_min_ (e Å^−3^)	0.62, −0.87

Computer programs: *APEX2* and *SAINT* (Bruker, 2015 ▸), *SHELXT* (Sheldrick, 2015*a* ▸), *SHELXL* (Sheldrick, 2015*b* ▸) and *Olex2* (Dolomanov *et al.*, 2009 ▸).

## supplementary crystallographic information

### Crystal data

**Table:**

C_9_H_7_Cl_2_N_5_·C_2_H_6_O	*F*(000) = 1248
*M**_r_* = 302.16	*D*_x_ = 1.420 Mg m^−^^3^
Monoclinic, *C*2/*c*	Mo *K*α radiation, λ = 0.71073 Å
*a* = 21.2458 (15) Å	Cell parameters from 7221 reflections
*b* = 10.2320 (8) Å	θ = 2.2–26.4°
*c* = 14.8428 (11) Å	µ = 0.46 mm^−^^1^
β = 118.808 (4)°	*T* = 100 K
*V* = 2827.3 (4) Å^3^	Block, colourless
*Z* = 8	0.39 × 0.25 × 0.13 mm

### Data collection

**Table:**

Bruker APEXII CCD diffractometer	2925 independent reflections
Radiation source: fine-focus sealed tube	2634 reflections with *I* > 2σ(*I*)
Graphite monochromator	*R*_int_ = 0.053
φ and ω scans	θ_max_ = 26.6°, θ_min_ = 2.2°
Absorption correction: multi-scan (SADABS; Bruker, 2015)	*h* = −26→26
*T*_min_ = 0.602, *T*_max_ = 0.745	*k* = −12→12
21376 measured reflections	*l* = −18→18

### Refinement

**Table:**

Refinement on *F*^2^	Primary atom site location: dual
Least-squares matrix: full	Hydrogen site location: inferred from neighbouring sites
*R*[*F*^2^ > 2σ(*F*^2^)] = 0.098	H-atom parameters constrained
*wR*(*F*^2^) = 0.234	*w* = 1/[σ^2^(*F*_o_^2^) + 59.8676*P*] where *P* = (*F*_o_^2^ + 2*F*_c_^2^)/3
*S* = 1.41	(Δ/σ)_max_ < 0.001
2925 reflections	Δρ_max_ = 0.62 e Å^−^^3^
193 parameters	Δρ_min_ = −0.87 e Å^−^^3^
48 restraints	

### Special details

**Table:**

Geometry. All esds (except the esd in the dihedral angle between two l.s. planes) are estimated using the full covariance matrix. The cell esds are taken into account individually in the estimation of esds in distances, angles and torsion angles; correlations between esds in cell parameters are only used when they are defined by crystal symmetry. An approximate (isotropic) treatment of cell esds is used for estimating esds involving l.s. planes.
Refinement. The occupancies of the disordered atoms in the ethanol were refined with their sum set to equal 1. Restraints were applied to maintain sensible thermal and geometric parameters. The diffraction data showed slight splitting of some peaks but twinning could not be sensibly separated and modelled. However this may explain the large K values, slightly high second weight paramater and Fobs greater than Fcalc.

### Fractional atomic coordinates and isotropic or equivalent isotropic displacement parameters (Å^2^)

**Table:**

	*x*	*y*	*z*	*U*_iso_*/*U*_eq_	Occ. (<1)
Cl1	0.16117 (9)	0.41684 (18)	0.10486 (12)	0.0254 (4)	
Cl2	0.06655 (8)	0.37527 (18)	0.21033 (13)	0.0256 (4)	
O1	0.4106 (2)	0.2925 (5)	0.4111 (3)	0.0192 (10)	
H1A	0.397808	0.357810	0.371958	0.029*	0.484 (14)
H1B	0.398027	0.358121	0.372284	0.029*	0.516 (14)
N3	0.3251 (3)	0.6534 (5)	0.1015 (4)	0.0136 (10)	
N4	0.2195 (3)	0.7095 (5)	0.0967 (4)	0.0145 (11)	
H4A	0.208617	0.757236	0.041803	0.017*	
H4B	0.189512	0.705023	0.121767	0.017*	
N1	0.3666 (3)	0.5147 (6)	0.2792 (4)	0.0177 (11)	
N2	0.4130 (3)	0.5288 (6)	0.2425 (4)	0.0185 (12)	
N5	0.4347 (3)	0.6032 (7)	0.1152 (4)	0.0293 (15)	
H5A	0.422053	0.646160	0.057650	0.035*	
H5B	0.477226	0.565850	0.147585	0.035*	
C6	0.2549 (3)	0.5427 (6)	0.2790 (4)	0.0144 (12)	
C8	0.2808 (3)	0.6445 (6)	0.1415 (4)	0.0133 (12)	
C7	0.3022 (3)	0.5648 (6)	0.2321 (4)	0.0142 (12)	
C4	0.2351 (4)	0.5649 (7)	0.4253 (5)	0.0191 (13)	
H4	0.251372	0.593442	0.493888	0.023*	
C3	0.1697 (3)	0.5029 (7)	0.3727 (5)	0.0193 (14)	
H3	0.140279	0.491009	0.404086	0.023*	
C2	0.1470 (3)	0.4581 (7)	0.2738 (5)	0.0176 (13)	
C9	0.3894 (3)	0.5948 (7)	0.1536 (5)	0.0189 (13)	
C5	0.2772 (3)	0.5859 (7)	0.3788 (5)	0.0188 (13)	
H5	0.321851	0.630185	0.415469	0.023*	
C1	0.1893 (3)	0.4782 (6)	0.2273 (5)	0.0152 (12)	
C10B	0.4570 (8)	0.2138 (16)	0.3909 (13)	0.024 (3)	0.484 (14)
H10A	0.435281	0.197369	0.316042	0.029*	0.484 (14)
H10B	0.464411	0.128524	0.426119	0.029*	0.484 (14)
C11B	0.5275 (8)	0.2819 (16)	0.4283 (14)	0.030 (4)	0.484 (14)
H11A	0.519935	0.365998	0.393000	0.045*	0.484 (14)
H11B	0.559689	0.227634	0.414026	0.045*	0.484 (14)
H11C	0.549146	0.296685	0.502588	0.045*	0.484 (14)
C10A	0.4866 (8)	0.2617 (17)	0.4439 (12)	0.030 (3)	0.516 (14)
H10C	0.500914	0.183860	0.489087	0.036*	0.516 (14)
H10D	0.516983	0.335764	0.484496	0.036*	0.516 (14)
C11A	0.5000 (8)	0.2356 (15)	0.3545 (12)	0.030 (3)	0.516 (14)
H11D	0.466604	0.168389	0.310075	0.045*	0.516 (14)
H11E	0.549503	0.205101	0.380438	0.045*	0.516 (14)
H11F	0.492715	0.316205	0.315082	0.045*	0.516 (14)

### Atomic displacement parameters (Å^2^)

**Table:**

	*U*^11^	*U*^22^	*U*^33^	*U*^12^	*U*^13^	*U*^23^
Cl1	0.0342 (9)	0.0289 (9)	0.0201 (8)	−0.0150 (7)	0.0189 (7)	−0.0098 (7)
Cl2	0.0144 (7)	0.0370 (10)	0.0260 (8)	−0.0077 (7)	0.0102 (6)	0.0040 (7)
O1	0.017 (2)	0.024 (2)	0.020 (2)	0.0066 (18)	0.0105 (18)	0.0100 (19)
N3	0.010 (2)	0.018 (3)	0.011 (2)	0.003 (2)	0.004 (2)	0.004 (2)
N4	0.011 (2)	0.020 (3)	0.012 (2)	0.005 (2)	0.006 (2)	0.007 (2)
N1	0.016 (3)	0.023 (3)	0.016 (3)	0.001 (2)	0.010 (2)	0.003 (2)
N2	0.010 (2)	0.031 (3)	0.015 (3)	0.006 (2)	0.007 (2)	0.008 (2)
N5	0.017 (3)	0.056 (4)	0.019 (3)	0.017 (3)	0.012 (2)	0.018 (3)
C6	0.015 (3)	0.016 (3)	0.013 (3)	0.005 (2)	0.008 (2)	0.004 (2)
C8	0.014 (3)	0.015 (3)	0.012 (3)	0.000 (2)	0.007 (2)	−0.001 (2)
C7	0.009 (3)	0.019 (3)	0.014 (3)	0.000 (2)	0.006 (2)	0.000 (2)
C4	0.025 (3)	0.020 (3)	0.013 (3)	0.003 (3)	0.010 (3)	0.003 (3)
C3	0.021 (3)	0.022 (3)	0.023 (3)	0.009 (3)	0.018 (3)	0.009 (3)
C2	0.014 (3)	0.021 (3)	0.019 (3)	−0.001 (3)	0.009 (3)	0.002 (3)
C9	0.015 (3)	0.027 (4)	0.017 (3)	0.008 (3)	0.010 (2)	0.008 (3)
C5	0.015 (3)	0.024 (3)	0.015 (3)	0.002 (3)	0.006 (2)	0.004 (3)
C1	0.019 (3)	0.015 (3)	0.012 (3)	0.003 (2)	0.009 (2)	0.002 (2)
C10B	0.020 (5)	0.026 (5)	0.028 (5)	0.006 (5)	0.013 (4)	0.005 (5)
C11B	0.020 (7)	0.028 (7)	0.044 (8)	0.008 (6)	0.017 (6)	0.015 (6)
C10A	0.017 (5)	0.039 (6)	0.031 (5)	0.007 (5)	0.011 (4)	0.013 (5)
C11A	0.030 (6)	0.027 (7)	0.040 (7)	0.009 (6)	0.021 (6)	0.008 (6)

### Geometric parameters (Å, º)

**Table:**

Cl1—C1	1.735 (6)	C8—C7	1.446 (8)
Cl2—C2	1.725 (6)	C4—H4	0.9500
O1—H1A	0.8400	C4—C3	1.377 (9)
O1—H1B	0.8400	C4—C5	1.386 (9)
O1—C10B	1.413 (15)	C3—H3	0.9500
O1—C10A	1.477 (14)	C3—C2	1.384 (9)
N3—C8	1.335 (7)	C2—C1	1.387 (8)
N3—C9	1.344 (8)	C5—H5	0.9500
N4—H4A	0.8800	C10B—H10A	0.9900
N4—H4B	0.8800	C10B—H10B	0.9900
N4—C8	1.322 (8)	C10B—C11B	1.50 (2)
N1—N2	1.345 (7)	C11B—H11A	0.9800
N1—C7	1.304 (8)	C11B—H11B	0.9800
N2—C9	1.346 (8)	C11B—H11C	0.9800
N5—H5A	0.8800	C10A—H10C	0.9900
N5—H5B	0.8800	C10A—H10D	0.9900
N5—C9	1.336 (8)	C10A—C11A	1.51 (2)
C6—C7	1.490 (8)	C11A—H11D	0.9800
C6—C5	1.392 (9)	C11A—H11E	0.9800
C6—C1	1.391 (9)	C11A—H11F	0.9800
			
C10B—O1—H1A	109.5	N5—C9—N2	116.5 (6)
C10A—O1—H1B	109.5	C6—C5—H5	119.7
C8—N3—C9	116.9 (5)	C4—C5—C6	120.6 (6)
H4A—N4—H4B	120.0	C4—C5—H5	119.7
C8—N4—H4A	120.0	C6—C1—Cl1	119.8 (5)
C8—N4—H4B	120.0	C2—C1—Cl1	119.2 (5)
C7—N1—N2	121.7 (5)	C2—C1—C6	120.9 (6)
N1—N2—C9	116.9 (5)	O1—C10B—H10A	109.8
H5A—N5—H5B	120.0	O1—C10B—H10B	109.8
C9—N5—H5A	120.0	O1—C10B—C11B	109.3 (13)
C9—N5—H5B	120.0	H10A—C10B—H10B	108.3
C5—C6—C7	119.2 (6)	C11B—C10B—H10A	109.8
C1—C6—C7	122.4 (5)	C11B—C10B—H10B	109.8
C1—C6—C5	118.3 (6)	C10B—C11B—H11A	109.5
N3—C8—C7	118.6 (5)	C10B—C11B—H11B	109.5
N4—C8—N3	118.5 (5)	C10B—C11B—H11C	109.5
N4—C8—C7	122.9 (5)	H11A—C11B—H11B	109.5
N1—C7—C6	117.3 (5)	H11A—C11B—H11C	109.5
N1—C7—C8	119.9 (5)	H11B—C11B—H11C	109.5
C8—C7—C6	122.7 (5)	O1—C10A—H10C	109.0
C3—C4—H4	119.8	O1—C10A—H10D	109.0
C3—C4—C5	120.5 (6)	O1—C10A—C11A	112.8 (12)
C5—C4—H4	119.8	H10C—C10A—H10D	107.8
C4—C3—H3	120.2	C11A—C10A—H10C	109.0
C4—C3—C2	119.6 (6)	C11A—C10A—H10D	109.0
C2—C3—H3	120.2	C10A—C11A—H11D	109.5
C3—C2—Cl2	119.3 (5)	C10A—C11A—H11E	109.5
C3—C2—C1	120.0 (6)	C10A—C11A—H11F	109.5
C1—C2—Cl2	120.7 (5)	H11D—C11A—H11E	109.5
N3—C9—N2	125.6 (5)	H11D—C11A—H11F	109.5
N5—C9—N3	117.9 (6)	H11E—C11A—H11F	109.5

### Hydrogen-bond geometry (Å, º)

**Table:**

*D*—H···*A*	*D*—H	H···*A*	*D*···*A*	*D*—H···*A*
O1—H1*A*···N1	0.84	2.01	2.848 (7)	179
N4—H4*A*···N3^i^	0.88	2.10	2.972 (7)	172
N4—H4*B*···O1^ii^	0.88	2.14	2.841 (7)	137
N5—H5*A*···O1^iii^	0.88	2.16	3.014 (7)	163
N5—H5*B*···N2^iv^	0.88	2.14	2.987 (8)	161

Symmetry codes: (i) −*x*+1/2, −*y*+3/2, −*z*; (ii) −*x*+1/2, *y*+1/2, −*z*+1/2; (iii) *x*, −*y*+1, *z*−1/2; (iv) −*x*+1, *y*, −*z*+1/2.
